# MPO-ANCA associated crescentic glomerulonephritis with numerous immune complexes: case report

**DOI:** 10.1186/1471-2369-13-32

**Published:** 2012-06-01

**Authors:** Ryuji Morizane, Konosuke Konishi, Akinori Hashiguchi, Hirobumi Tokuyama, Shu Wakino, Hiroshi Kawabe, Matsuhiko Hayashi, Koichi Hayashi, Hiroshi Itoh

**Affiliations:** 1 Department of Internal Medicine, Keio University School of Medicine, 35 Shinanomachi, Shinjuku-ku, Tokyo 160-8582, Japan; 2 Department of Pathology, Keio University School of Medicine, 35 Shinanomachi, Shinjuku-ku, Tokyo 160-8582, Japan; 3 Health Center, Keio University, 35 Shinanomachi, Shinjuku-ku, Tokyo 160-8582, Japan; 4 Apheresis and Dialysis Center, Keio University School of Medicine, 35 Shinanomachi, Shinjuku-ku, Tokyo 160-8582, Japan

**Keywords:** Myeloperoxidase-antineutrophil cytoplasmic antibody, Membranoproliferative glomerulonephritis, Immune complex, Rapidly progressive glomerulonephritis

## Abstract

**Background:**

Antineutrophil cytoplasmic antibody (ANCA)-associated crescentic glomerulonephritis (CGN) is a major cause of rapidly progressive glomerulonephritis (RPGN). ANCA-associated CGN is generally classified into pauci-immune RPGN, in which there are few or no immune complexes.

**Case Presentation:**

A 78-year-old man presented with RPGN after a 7-year course of chronic proteinuria and hematuria with stable renal function. A blood examination showed a high titer of myeloperoxidase (MPO)-ANCA. A renal biopsy showed crescentic glomerulonephritis with abundant subepithelial, intramenbranous and subendothelial deposits by electron microscopy, leading to the diagnosis of ANCA-associated CGN superimposed on type 3 membranoproliferative glomerulonephritis (MPGN).

**Conclusions:**

This case is unique in that type 3 MPGN and MPO-ANCA-associated CGN coexisted, and no similar case has been reported to date. Because ANCA-associated CGN has a predilection for elderly individuals and primary type 3 MPGN is rarely seen in this age group, coincidental existence appears less likely. This case may confer valuable information regarding the link between immune complex and ANCA-associated CGN.

## Background

In elderly people, myeloperoxidase (MPO) and proteinase-3 (PR3) antineutrophil cytoplasmic antibody (ANCA)-associated crescentic glomerulonephritis (CGN) is a major cause of rapidly progressive glomerulonephritis (RPGN). ANCA-associated CGN is generally classified into pauci-immune RPGN, in which there are few or no immune complexes. We have experienced a rare case of ANCA-associated CGN with extensive glomerular immune deposits supposed as type 3 membranoproliferative glomerulonephritis (MPGN). Since idiopathic MPGN is rarely seen in elderly subjects, coincidence of ANCA-associated CGN and MPGN appears less likely. Here, we present a case showing quite unique pathological findings and further discuss the possible association between ANCA-associated CGN and immune complex disease.

## Case presentation

A 78-year-old Japanese man was admitted to our hospital because of rapidly declining renal function. The patient had been treated for hypertension since the age of 63 years at our hospital and was initially free of proteinuria with normal renal function. Despite satisfactory control of hypertension, he began to exhibit proteinuria with microscopic hematuria at the age of 71 years. Proteinuria gradually increased to 2 grams per day over the ensuing six months. Laboratory and imaging studies for connective tissue disease, malignancy, dysproteinemia, and hepatitis viral infection were uninformative, but a test for antinuclear antibodies was weakly positive and a high-resolution computed tomography scan showed mild interstitial pneumonia. Although a renal biopsy was recommended at this time, the patient did not consent to the procedure, but received antihypertensive treatment under the close observation. Proteinuria and microscopic hematuria persisted, and his serum creatinine remained at the level of 1.2 to 1.4 mg/dL. Seven years after the onset of the proteinuria, however, the patient’s serum creatinine level rose rapidly from 1.4 mg/dL to 8.1 mg/dL over a period of 2 months, and he was admitted to our hospital.

The patient was 167.5 cm tall and weighed 64.7 kg. His blood pressure was 142/77 mmHg. His body temperature was 35.8°C. The palpebral conjunctivae showed pallor, and marked edema was present in the lower extremities. No respiratory or neurologic abnormalities were apparent.

Laboratory data are shown in Table 1. Serum creatinine was markedly increased (i.e., 8.3 mg/dL). A urinalysis showed massive proteinuria (i.e., 3+), and the sediment contained 50 to 100 red blood cells/high power field as well as granular and waxy casts. 24-hour urinary protein excretion was 3.17 g. Serologic evaluation revealed the presence of ANCA directed against myeloperoxidase (MPO-ANCA) at 536 EU (normal, < 20 EU). The computed tomography scan of the lung showed interstitial pneumonia with reticular and ground glass opacity, predominantly in the peripheral lower lung accompanied by a honeycomb appearance. Bilateral apical old inflammatory changes suggestive of healed tuberculosis were also present.

**Table 1:** Laboratory data on admission

			
white blood cell count	8,900/mm^3^	MPO-ANCA	536 EU (normal, < 20 EU)
hemoglobin	8.7 g/dL	PR3-ANCA	negative
plate let count	343,300/mm^3^	antinuclear antibody	+/-
creatinine	8.3 mg/dL	anti-DNA antibody	negative
urea nitrogen	65.1 mg/dL	anti-SSA antibody	negative
total protein	5.2 g/dL	anti-SSB antibody	negative
albumin	1.5 g/dL	anti-Sm antibody	negative
total cholesterol	147 mg/dL	anti-U1RNP antibody	negative
sodium	135.4 mEq/L	anti-GBM antibody	negative
potassium	5.2 mEq/L	rheumatoid factor	negative
bicarbonate	15.4 mg/dL	hepatitis B antigen	negative
calcium	7.6 mg/dL	hepatitis c antigen	negative
phosphorus	7.3 mg/dL	KL-6	337 U/ml (normal, <500 U/mL
aspartate amino transferase	25 IU/L	surfactant protein D	77 ng/mL (normal, 110 ng/mL)
alanine amino transferase	13 IU/L	surfactant protein A	56.5 ng/mL (normal, <43.8 U/mL
C3	71 mg/dL (normal, 60-116 mg/dL)	QuantiFERON®	nagative
C4	42 mg/dL (normal, 15-44 mg/dL)		
CH-50	49.4 U/mL (normal, 25.0-48.0 mg/dL)		

**Figure 1:**
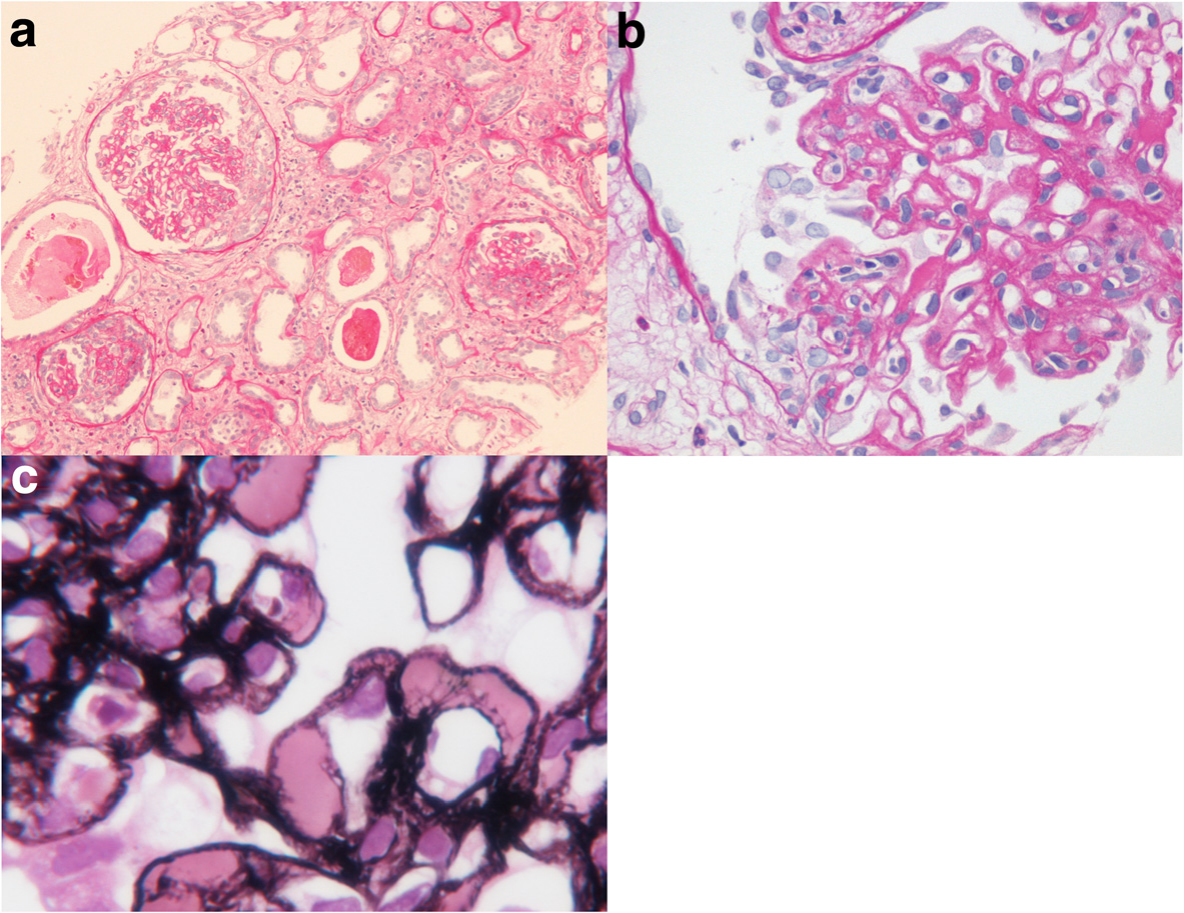
**Light microscopy findings.a)** Several crescents with focal endocapillary hypercellularity are visible in the glomeruli (Periodic acid-Schiff, ×200 original magnification). **b)** Glomeruli are showing endocapillary and extracapillary hypercellularity. The capillary walls are diffusely thickened. **c)** Membranous deposits continuous with large mesangial and subendothelial deposits. The glomerular basement membrane shows numerous spikes and reticulation (Methenamine sliver, ×1,000 original magnification).

**Figure 2:**
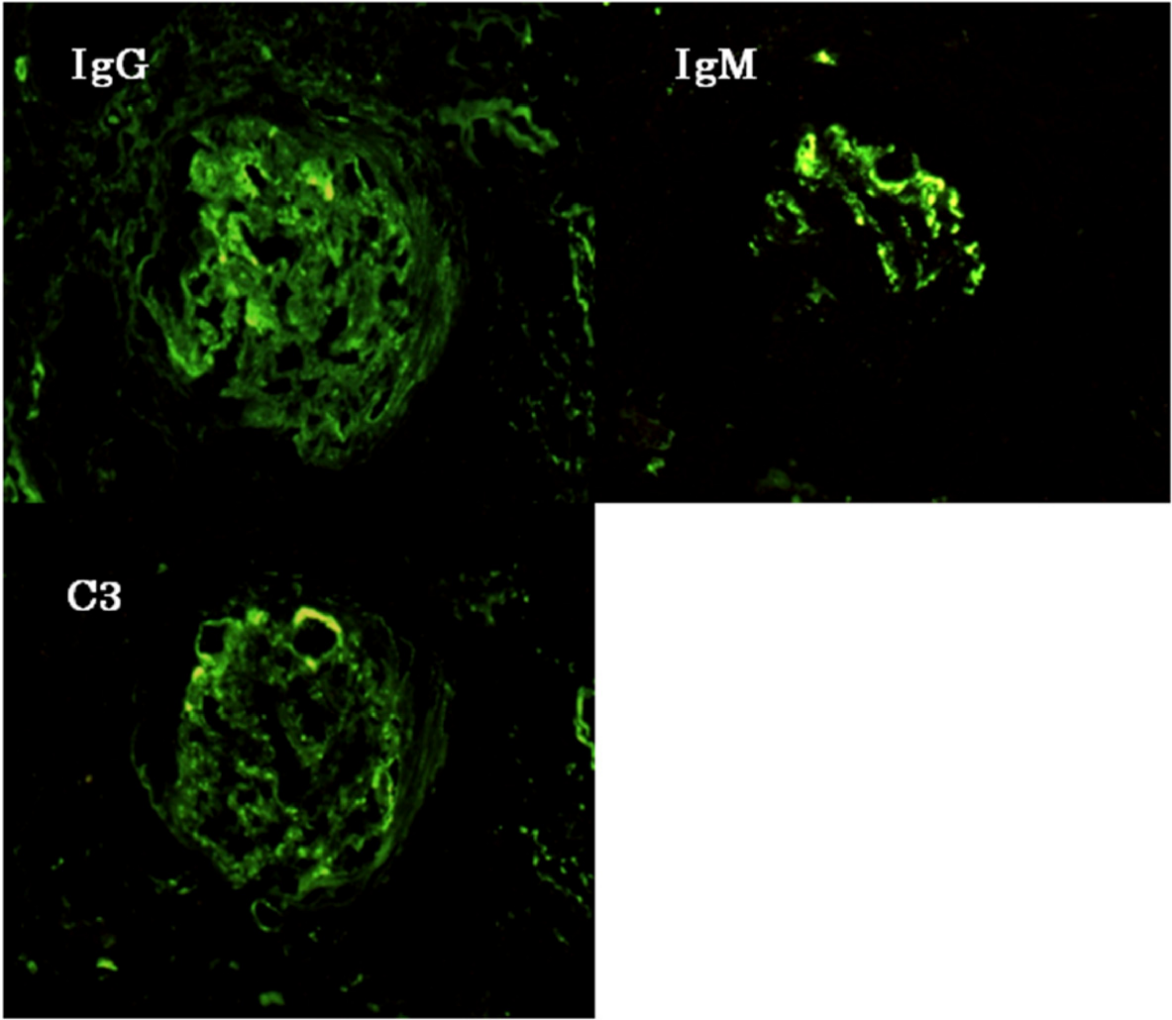
** Immunofluorescence microscopy findings.** Immunofluorescent studies showed heavy, granular, epimembranous deposits that stained positive with antisera directed against IgG, IgM, and C3.

**Figure 3:**
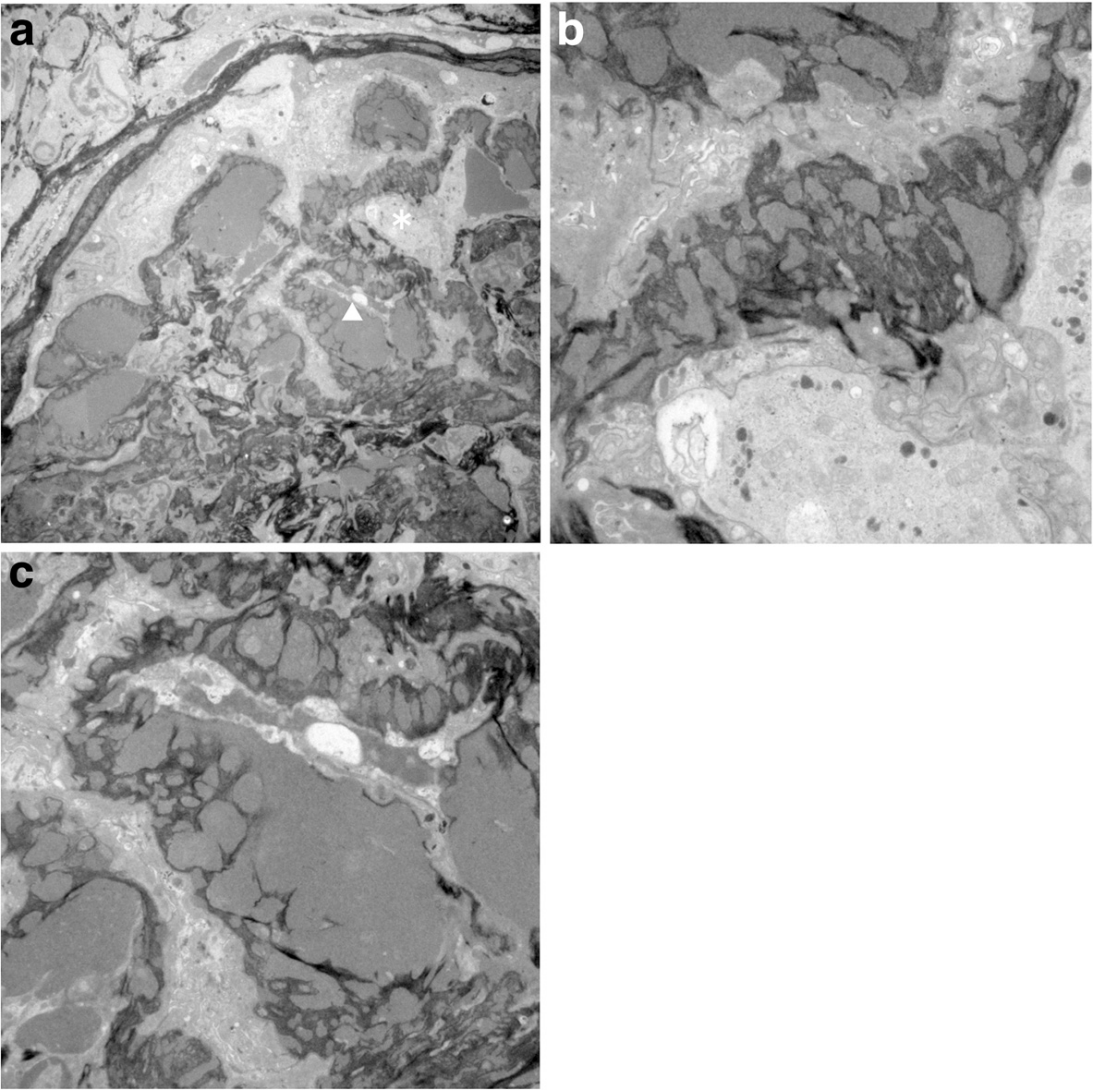
** Silver-impregnated specimens observed using electron microscopy. a)** Low magnification (×300). The portion indicated by an asterisk and an arrowhead are enlarged to **b)** and **c)** respectively. **b)** Numerous large intra- and trans-membranous electron-dense deposits are visible (original magnification, ×1,000). **c)** Glomerular basement membrane shows a disrupted, reticulated and woven appearance (original magnification, ×1,500).

Because of the rapidly declining renal function, hemodialysis was started shortly after admission. A renal biopsy was performed on the 7th hospital day. Renal histological findings were as follows; six of the nine glomeruli visible by light microscopy showed cellular and/or fibrocellular crescents with focal endocapillary hypercellularity (Figure 1). The capillary walls were diffusely thickened. Diffuse epimembranous deposits that were occasionally continuous with large mesangial and subendothelial deposits were observed. Arteries and arterioles were free of vasculitic changes. Immunofluorescent studies revealed heavy, granular deposits of the capillary walls that stained positive with antisera directed against IgG, IgM, and C3 (Figure 2). Silver-impregnated samples observed using electron microscopy showed diffuse glomerular basement membrane (GBM) thickening and reticulation with large intra- and trans-membranous deposits. The deposits were not concentrated in the lamina densa, as was observed in dense-deposit disease (Figure 3).

The patient was treated with high-dose prednisolone for 6 weeks following 3 days of intravenous pulse methylprednisolone treatment. Cyclophosphamide was not administered when his age and the risk of infection were deliberately taken into consideration. Although the MPO-ANCA titer decreased with steroid therapy, the patient’s renal function failed to improve and prednisolone was tapered. The patient was subsequently discharged from the hospital and continued to receive maintenance hemodialysis therapy on an outpatient basis.

## Discussion

Our patient presented with a clinical picture of RPGN, with a high serum titer of MPO-ANCA preceded by seven years of proteinuria and microscopic hematuria with mildly compromised but stable renal function. Renal biopsy showed diffuse glomerular crescent formation as well as abundant deposits and diffusely thickened capillary walls by light microscopy. Because the co-existence of membranous nephropathy and ANCA-associated CGN was shown in previous reports [1-5], we inferred a priori that MPO-ANCA-associated CGN was superimposed on membranous nephropathy. Electron microscopic observation, however, revealed not only subepithelial but also numerous intramembranous and subendothelial deposits, which are rarely observed in membranous nephropathy. These findings rather favor a diagnosis of MPO-ANCA-associated CGN superimposed on type 3 MPGN, although mesangial proliferation was only modest. Type 3 MPGN, however, is rarely seen in elderly people, and no reports have previously been made showing ANCA-associated CGN superimposed on type 3 MPGN.

It has been widely accepted that the paucity of immunoglobulin deposits distinguishes ANCA-associated CGN from immune complex glomerulonephritis and anti-GBM glomerulonephritis, leading to the belief that immune complexes do not play a role in ANCA-associated CGN. Nevertheless, several studies suggest that glomerular immune complex deposition and ANCA-positivity are not necessarily mutually exclusive. Falk et al. [6] demonstrated that a considerable number of patients with immune complex glomerulonephritis were positive for ANCA. Furthermore, ANCA is reported to be detected in various immune complex-mediated nephropathy, including IgA nephropathy [7,8], post-infectious glomerulonephritis [9], membranous nephropathy [5], lupus nephritis [10-12], glomerulonephritis of hypocomplementemic urticarial vasculitis syndrome [13], glomerulonephritis of hepatitis C virus infection [14,15] and glomerulonephritis of subacute bacterial endocarditis [16]. These observations suggest a possible link between immune complex and ANCA in a subset of glomerulonephritis.

Alternatively, Neumann et al. [17] evaluated 45 patients with systemic small vessel vasculitis and CGN or idiopathic CGN, and found substantial immunoglobulin deposition in 8 of 45 cases (18%), using the immune-peroxidase method. Haas et al. [9] also demonstrated the presence of electron-dense deposits in 68 cases (54%) among 126 patients of CGN associated with ANCA and/or necrotizing angiitis. Finally, in a study in which renal biopsy was performed in 74 patients with primary ANCA-associated systemic vasculitis, 23 cases (31%) possess immune complex deposition, as assessed by immunofluorescence and/or electron microscopy [18]. In concert, the presence of glomerular immune complex deposition does not preclude the presence of ANCA or ANCA-associated CGN.

Our case has manifested clinical and pathological features of MPO-ANCA-associated CGN with numerous immune deposits, which is compatible with the diagnosis of MPO-ANCA-associated CGN superimposed on idiopathic type 3 MPGN. It is well known that ANCA-associated CGN has a predilection for elderly individuals whereas primary type 3 MPGN is rarely seen in this age group. Coincidental occurrence of the two conditions therefore appears less likely. Alternatively, several studies reveal positive ANCA in secondary MPGN (Table 2). Constantin et al. [19] reported a patient of rheumatoid arthritis with positive p-ANCA and type 1 MPGN. Furthermore, Bonarek et al. [20] experienced a case of infected cysto-atrial shunt with c-ANCA-positive type 1 MPGN. Similarly, Lhotta et al. [21] reported a patient with carbamazepine-induced cryoglobulinemia and p-ANCA-positive MPGN. In our present case, however, we were unable to disclose any distinct disease condition that could cause these two types of renal pathological changes. In this regard, our patient manifests interstitial pneumonia and positive antinuclear antibody, which prompts us to surmise that this case has deranged autoimmune system that could cause MPGN and ANCA-associated CGN. Of interest, a couple of previous animal experiments showed a role of immune complexes in the pathogenesis of ANCA-associated CGN [22,23]. It requires further basic and clinical studies to elucidate whether immune complex-mediated diseases induce ANCA-associated CGN. 

**Table 2:** Cases of secondary MPGN with positive ANCA

**Diagnosis**	**Type of ANCA**	**Clinical course**	**Renal pathology**	**Reference**
Rheumatoid arthritis	p-ANCA 1:1000	nephrotic syndrome	type 1 MPGN with crescent IgG/M/C1q/C3 in subendothelium	[19]
shunt nephritis	c-ANCA 1:1000 ELISA: 39 IU/ml	proteinuria (2.1 g/day)	type 1 MPGN IgM/A/C3 in subepithelial	[20]
carbamazepine induced autoimmune	p-ANCA 1:1280	RPGN	type 1 MPGN with crescent IgG/M/C1q/C3 in subendothelium	[21]

## Conclusions

Herein, the authors present a case with unique pathological changes showing a possible link between ANCA-associated nephropathy and immune complex. The implication that immune complex-mediated glomerular injury plays a pathogenetic role in a subset of ANCA-associated crescentic glomerulonephritis warrants further investigations.

## Consent

Written informed consent was obtained from the patient for publication of this case report and any accompanying images. A copy of the written consent is available for review by the Editor-in-Chief of this journal.

## Competing interests

The authors declare that they have no competing interests.

## Authors’ contributions

RM treated the patient as an inpatient and made decisions about patient's examinations and therapies. RM reviewed previous publications and wrote the whole manuscript. KK is a director of renal pathology in our hospital. KK read patient's renal pathology and supervised the manuscript. AH is a pathologist in our hospital. AH made sections of all renal biopsy samples, and read patient's renal pathology. AH took pictures of renal pathology and wrote the pathological findings in the manuscript. HT and SW are nephrologist in our hospital, and performed renal biopsy of the patient. They also read the renal pathology. HK was a principal physician of the patient and treated him as an outpatient. MH is a professor of dialysis center in our hospital. MH treated the patient by hemodialysis. KH is a associate professor of nephrology, and supervised the manuscript. HI is a professor of nephrology, endocrinology and metabolism, and supervised the manuscript. All authors read and approved the final manuscript.

## Pre-publication history

The pre-publication history for this paper can be accessed here:

http://www.biomedcentral.com/1471-2369/13/32/prepub
